# An Experimental and Computational Analysis of Plant Compounds from Whole *Urtica dioica* L. Plant’s Essential Oil for Antioxidant and Antibacterial Activities

**DOI:** 10.3390/metabo13040502

**Published:** 2023-03-30

**Authors:** Muhammad Zahid Khan, Abul Kalam Azad, Saleem Jan, Muhammad Safdar, Shabana Bibi, Amin Malik Shah Abdul Majid, Ghadeer M. Albadrani, Nehal Ahmed Talaat Nouh, Jawaher A. Abdulhakim, Mohamed M. Abdel-Daim

**Affiliations:** 1 Department of Chemistry, University of Science and Technology, Bannu 28100, Pakistan; 2 Faculty of Pharmacy, MAHSA University, Bandar Saujana Putra, Jenjarom 42610, Selangor, Malaysia; 3 Department of Pharmaceutics, Faculty of Pharmacy, Gomal University, Dera Ismail Khan 29050, Pakistan; 4 Department of Biosciences, Shifa Tameer-e-Millat University, Islamabad 44000, Pakistan; 5 Yunnan Herbal Laboratory, College of Ecology and Environmental Sciences, Yunnan University, Kunming 650091, China; 6 Eman Research Ltd., 10-14 Wormald St, Symonston, ACT 2609, Australia; 7 Department of Biology, College of Science, Princess Nourah bint Abdulrahman University, Riyadh 11671, Saudi Arabia; 8 Department of Microbiology, Medicine Program, Batterjee Medical College, P.O. Box 6231, Jeddah 21442, Saudi Arabia; 9 Inpatient Pharmacy, Mansoura University Hospitals, Mansoura 35516, Egypt; 10 Medical Laboratory Department, College of Applied Medical Sciences, Taibah University, Yanbu 46522, Saudi Arabia; 11 Department of Pharmaceutical Sciences, Pharmacy Program, Batterjee Medical College, P.O. Box 6231, Jeddah 21442, Saudi Arabia; 12 Pharmacology Department, Faculty of Veterinary Medicine, Suez Canal University, Ismailia 41522, Egypt

**Keywords:** *Urtica dioica* L. essential oil, GC–MS, phytochemical analysis, antioxidant activities, antibacterial activities

## Abstract

As the *Urtica dioica* L. whole plant’s essential oil has presented significant multiple activities, it was therefore evaluated using the GC–MS technique. This essential oil was investigated for its antioxidant, phytotoxic, and antibacterial activities in vitro. The GC–MS analysis data assisted in the identification of various constituents. The study of the essential oil of *U. dioica* showed potential antioxidant effects and antibacterial activity against the selected pathogens *Escherichia coli* -ATCC 9837 (*E. coli*), *Bacillus subtilis*-ATCC 6633 (*B. subtilis*), *Staphylococcus aureus*-ATCC6538 (*S. aureus*), *Pseudomonas aeruginosa*-ATCC 9027 (*P. aeruginosa*), and *Salmonella typhi*-ATCC 6539 (*S. typhi*). The library of 23 phytochemicals was docked by using MOE software, and three top virtual hits with peroxiredoxin protein [PDB ID: 1HD2] and potential target protein [PDB ID: 4TZK] were used; hence, the protein–ligand docking results estimated the best binding conformations and a significant correlation with the experimental analysis, in terms of the docking score and binding interactions with the key residues of the native active binding site. The essential oil in the silico pharmacokinetic profile explained the structure and activity relationships of the selected best hits, and their additional parameters provided insight for further clinical investigations. Therefore, it is concluded that the *U. dioica* essential oil could be a potent antioxidant and antibacterial agent for aromatherapy through its topical application, if further tested in a laboratory and validated.

## 1. Introduction

The genus *Urtica* is associated with the family “Urticaceae” in the division of Angiosperms, which present peculiar flowering characteristics which have been known for a long time, and are also utilized as traditional medicine and are significant as food [1]. The closest associates of this genus are the stinging nettle *Urtica dioica* L. (*U. dioica*) and the small nettle *U. urens* L., which are native to Asian, African, European, and North American areas [2]. *U. dioica* L., commonly known as the common nettle, is a persistent plant that grows in moderate and humid wilderness ranges across the world [3]. It possesses pointed leaves and grows from 1 to 1.4 m high. The isolation, purification, and characterization of antimicrobial plant-derived compounds remains a stimulating research field, particularly in the drug development field for multi-drug resistant bacteria [4,5].

*U. dioica* is extensively recycled as a traditional medicine for the management of allergies [6], stone formation, anemia, rashes [7], hypertension [8], antiarthritic and antirheumatic effects [9], antidandruff, galactagogue, hemostatic, and anti-ulcer activity, stomachache and liver dysfunction, and anti-inflammatory [10,11], antihyperglycemic [10], antioxidant, acute diuretic, natriuretic, and hypotensive effects [12]. It has been used in the traditional therapy of antifungal agents [13].

The chemical constituents of the *U. dioica* plant also exhibit anticancer and anti-diabeties mellitus (anti-DM) properties. Existing knowledge suggests that *U. dioica* possesses fatty acids, sterols, lignans, carotenoids, plastocyanins, glycoproteins, lectins, polysaccharides, terpenes, and flavonoids as its main phytoconstitutents [14]. The identification of essential oils is an interesting component of phytochemical investigation [15,16,17].

As the plant *U. dioica* in its crude form and its various constituents exhibited biological potential, we intended to perform a gas chromatography–mass spectrometry (GC–MS) analysis of *U. dioica*’s essential oil, and to evaluate its antioxidant, phytotoxic, and anti-bacterial potential. For the validation of the experimental results, we followed in silico techniques, such as molecular docking (MD) and ADMET, to highlight each ligand’s behavior with respect to specific selected proteins.

In this study, the *U. dioica* plant’s oil revealed potential antioxidant and antimicrobial activities, potentially for first time while using GC–MS-derived bioactive compounds from the *U. dioica* plant’s oil. The most significant outcome of this study was to reveal the phytotoxic effect of the oil for the first time. In this study, another objective was to accomplish the docking interaction of the compounds with proteins, using in silico methods and mutagenesis experiments, which are in the pipeline to be performed in upcoming studies. 1HD2 is an extensively studied antioxidant enzyme, and 4TZK is used in several studies to test antibacterial effects.

## 2. Materials and Methods

### 2.1. Sampling

Our research was conducted to collect samples in a suitable season from a specific area, where the plant growth is good and available. According to the sampling plans, the whole *U. dioica* plant was collected in March 2019 from the Dardarez Landidak regions of Bannu (Latitude: 32°59′9.9996″ N and Longitude: 70°36′14.9904″ E), KPK, Pakistan. The plant was identified by Dr. Faizan Ullah, Assistant Professor, Department of Botany, University of Science and Technology Bannu, Bannu, Pakistan.

### 2.2. Extract Preparation

The whole plant material was dried in a shady area, ground well, and then soaked in 80% aq. methanol (MeOH) for one week. The soaked material was evaporated by using a rotary evaporator to obtain a dark, gummy residue. The gummy material was initially extracted with n-hexane to remove the fatty materials. The defatted MeOH extract was suspended in water, and then the aqueous fraction was further fractionated with dichloromethane (DCM), ethyl acetate (EtOAc), and butanol. The DCM fraction was preceded further by column chromatography and the essential oil part was passed through gas chromatography (GC) and gas chromatography mass spectrum (GC–MS) instruments, and the components were identified.

### 2.3. Gas Chromatography–Mass (GC–MS) Spectrum Analysis

The essential oil component of the dichloromethane fraction of the *U. dioica* was analyzed through a GC–MS Agilent 6890N Network GC system, combined with an Agilent 5973 Network Mass Selective Detector (GC–MS) instrument, under computer control. The injector temperature was set at 220 °C for 5 min. At a split ratio of 1:10. 1 µL, a volume of 1000 ppm of the essential oil solution (GC Grade n, hexane, scharlau, chemia, Barcelona, Spain) was injected. Initially, the column was maintained at 50 °C for 2 min and then increased to 150 °C, at which it was held isothermal for 5 min, and a second rmp (200 per minutes) was applied to 220 °C and held isothermal for 10 min. The total run time was 120 min. Thus, it was maintained between 180 °C and 230 °C, respectively. The MS was performed in the electron ionization mode (70 eV) [18].

### 2.4. Antioxidant Activity

The antioxidant activity was estimated with a DPPH assay [19,20]. The DPPH solution was set via dissolving 3.2 mg in 100 mL of 82% methanol, and then 2.8 mL of the DPPH solution was added to the glass vial and monitored with the addition of 0.2 mL of the test sample solution, leading to the final concentration of 1 µg/mL, 5 µg/mL, 10 µg/mL, 25 µg/mL, 50 µg/mL, and 100 µg/mL. Ascorbic acid was used as a standard. The process was performed under dark conditions at fixed room temperature for 1 h, the discoloration was measured at 517 nm in triplicate by using a UV spectrophotometer (Deuterium lamp, Shimadzu, Japan), and the radical scavenging capacity was expressed as a percentage effect (E%) and estimated by using the following equations.

$$\%Scavenging\;=\;\frac{Absorption\;of\;control-absorption\;of\;fraction}{Absorption\;of\;control}\;\times \;100$$

### 2.5. Phytotoxic Activity

The phytotoxic activity was estimated with the application of a modified protocol of *Lemna minor* L. [15]. Inorganic E-medium was prepared by mixing the appropriate inorganic constituents into 1 L of distilled water, and the pH was accustomed at 5.5–5.6 by adding KOH solution and autoclaved at 121 °C for 15 min. The essential oil served as a stock solution. A total of three sterilized flasks for each concentration were inoculated with 1000 µL, 100 µL, and 10 µL of the stock solution for 500, 50, and 5 ppm, respectively. Each flask, medium (20 mL), and essential oil, each encompassing a rosette of three fronds of the *Lemna minor* L., were added. In total, two supplemented flasks, one with the standard drug (Paraquat) and the other with the E-medium, were served as positive and negative controls, respectively. All the flasks were wrought with cotton and were reserved in the growth cabinet for seven days. The number of fronds per flask was calculated and noted at day seven. The results were interpreted by considering the growth regulation as a percentage and considered with reference to the negative control.

(1) $$\%Growth\;regulation\;=\;100\;-\;\frac{Number\;of\;fronds\;in\;test\;sample}{Number\;of\;fronds\;in\;negative\;control}\;\times \;100$$

### 2.6. Antibacterial Activity

To understand the antibacterial effect, Microplate Alamar Blue Assay was used [21,22]. Mueller–Hinton medium was used for the growth of the organism. This is a non-selective, non-differential, microbiological growth medium. It contained beef extract, acid casein hydrolysate, starch, and agar. The beef extract and casein acid hydrolysate provided nitrogen, vitamins, carbon, amino acids, sulfur, and other essential nutrients. The inoculation adjustment was done to a 0.5 McFarland turbidity index. The essential oil samples were mixed in pure DMSO with a 1:1 ratio [23]. The media were poured into all the wells that did not contain any test samples. The reaction mixture was a total of 200 µL and 96-well microtiter plates were used. In the end, about 5 × 10^6^ cells were added in each well of the controls, as well as the tests. The plate was wrapped with paraffin film and was incubated for about 20 h. After incubation, the suggested dye, Alamar Blue, was distributed in each well, and the plate was well shaken at 80 RPM in an incubator with a shaking mode for about 3 h. The plate was covered with aluminum and essential oil while the shaking was carried out in the shaking incubator. The color of the Alamar Blue dye changing from blue to pink showed the growth of certain bacterial strains such as *Escherichia coli* -ATCC 9837 *(E. coli)*, *Bacillus subtilis*-ATCC 6633 (*B. subtilis*), *Staphylococcus aureus*-ATCC6538 (*S. aureus*), *Pseudomonas aeruginosa*-ATCC 9027 (*P. aeruginosa*), and *Salmonella typhi*-ATCC 6539 (*S. typhi*). The absorbance of each well was monitored in the ELISA reader at a 570–600 nm wavelength.

### 2.7. Computational Analysis

#### 2.7.1. Selection of Protein Targets and Chemical Compounds

In pharmaceutical research and computer-aided drug design (CADD), one of the most significant computational techniques is called MD [24,25]. The fundamental requirement of protein–ligand MD protocols is to find the probable binding geometries of a suspected ligand with a known three-dimensional structure and a target protein [26]. This may be accomplished by comparing the structures of both the hypothetical ligand and the targeting protein. The 2D structures of the phytochemical dataset were sketched with the ChemDraw application for further analysis [27] (Table 1). In this study, we targeted the peroxiredoxin protein [PDB ID: 1HD2] [28] and the potential selected macromolecule [PDB ID: 4TZK] [29] for the MD analysis, in order to check its correlation with the experimental analysis of the whole *Urtica dioica* L. plant’s essential oil compounds.

#### 2.7.2. Molecular Docking (MD) and Interaction Analysis

MD is a suitable molecular modeling procedure, which is significantly based on the searching and ranking of poses to generate an MD score scheme, and the best binding configurations that are estimated from a best-docked complex, which help to explore the binding interactions of a protein–ligand; therefore, it could be useful to understand the molecular functions of a ligand that is confined within the active residual region of the selected enzyme [30,31]. For MD purposes, 5 compounds were used, and the selected proteins’ three-dimensional structures (PDB ID: 1HD2 and 4TZK), in .pdb format, were downloaded from the PDB and prepared in a molecular operating environment (MOE) software [32,33]. The water molecules from the ligand that was already bound to the selected enzyme molecule were detached, and for the heteroatoms, 3D protonation was performed to prepare its structure for the MD procedure. In each protein structure, an active site was acknowledged, and PRO40, THR44, PRO45, LEU116, PHE120, GLY46, CYS47, ARG127, and THR147 residues were selected for the active residual region of the 1HD2 protein, while GLY14, ILE15, ILE16, ALA22, PHE41, ALA190, ALA191, GLY192, PRO193, ILE194, MET147, ASP148, PHE149, MET155, VAL65, GLN66, THR17, SER20, ILE21, HIS93, SER94, ILE96, GLY97, PHE98, MET99, MET103, LEU63, ASP64, GLY104, ILE122, PRO156, ALA157, TYR158, MET161, LYS165, THR196, ALA198, MET199, ILE202, LEU207, ALA211, ILE215, and LEU218 residues were selected for the active site of the 4TZK protein. A structural optimization was also applied by notable estimations, such as the addition of hydrogen atoms and an Amber14 force field scheme, and a realistic scheme with chiral constraints was applied, as were geometrical constraints for extra energy control of the potent bounded conformers. The surfaces and maps panel module was used to manage the surface’s structural transparency and resulted in evidence of important amino acids in the selected region of the enzyme selected as the initial deigned conformer. A database of 23 phytochemicals that was retrieved from the experimental analysis was created in the MOE software, in order to perform MD simulations, and the database was saved with an .mdb extension for next-level analysis. The enhancement and estimations of binding-free energies (ΔG) were evaluated with the application of a scoring function (GBVI/WSA dg) to screen the top-ranked poses that were performed [34]. Pi, hydrogen, and hydrophobic interactions as the consistent scoring pattern were established in the form of an MD score of the correct binding poses [35]. The database of the docked complex, which was generated in the MOE, was visualized for a detailed understanding of the mode of the binding interactions of the ligands in the selected active site of the target protein.

#### 2.7.3. Pharmacokinetic/ADMET Profile Estimation

The selected best-docked compounds were used for the estimations of the ADMET (absorption, distribution, metabolism, excretion, and toxicity) properties in order to justify the drug-like assumptions, as this is considered to be an essential criterion for the drug-like screening of chemical libraries [24,36]. For the purpose of the ADMET profile estimation of the selected potential hits, SwissADME [37] and Datawarrior tools [38] were used.

## 3. Results and Discussion

**Figure 1:**
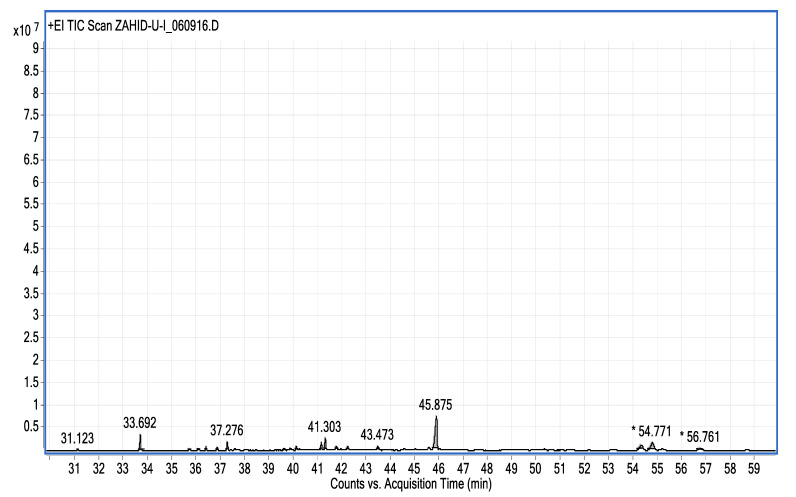
GC–MS chromatogram showing peaks in the range of 31–59, * denoted high values.

**Figure 2:**
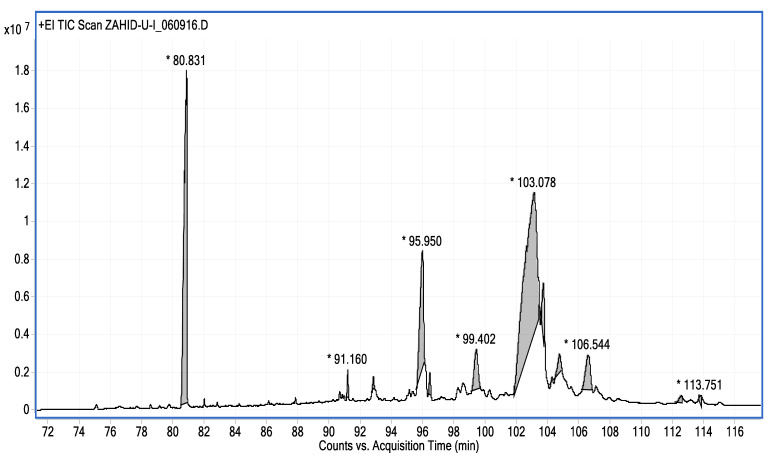
GC–MS chromatogram showing peaks in the range of 72–116, * denoted high values.

The study showed a GC–MS analysis of the isolated essential oils of *U. dioica (*Figure 1 and Figure 2). The GC–MS chromatogram showed various peaks, which, upon comparison with data from the literature, were identified as nonanoic acid, 9-oxo- ethyl ester, caryophyllene oxide, limonen-6-ol-pivalate, β-himachalenoxid, 4-tert-butyltoluene, α-bisabolol, cholestan-3-ol,2-methylene-, (3β, 5α), benzenepropanol 2,4,6-trimethyl, Z-(13,14-epoxy)tetradec-11-en-1-ol acetate, hexadecanoic acid, ethyl ester, 3,7,11,15-tetramethyl-2-hexadecen-1-ol, 2-pentadecanone, 6,10,14-trimethyl, hexadecanoic acid, methyl ester, 9,12-octadecadienoic acid, ethyl ester, ethyl 9,12,15-octadecatrienoate, 1,2-benzenedicarboxylic acid, mono(2-ethylhexyl) ester, 16-hentriacontanone, Z-5-methyl-6-heneicosen-11-one, 18-pentatriacontanone, E,E,Z-1,3,12-nonadecatriene-5,14-diol, 9-octadecenoic acid (Z), 9-octadecenyl ester, (Z), and tricyclo [20.8.0.0(7,16)] triacontane, 1(22),7(16)-diepoxy. The identification of these compounds was made by the comparison of their retention time and the mass spectra of those stored in the computer library and published literature. The mass, retention time, % area, molecular formula, fragment ions, and their structures, are hereby summarized in Table 1.

**Figure 3:**
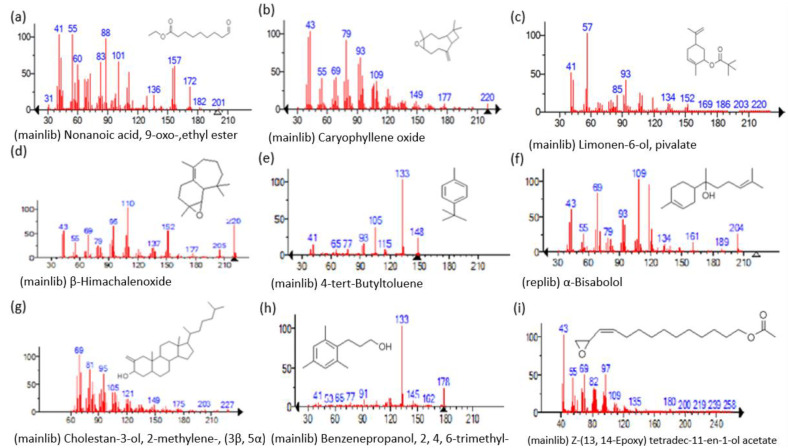
Representing the mass spectrum of Nonanoic acid, 9-oxo-, ethyl ester with RT= 33.692 (**a**), Caryophyllene oxide with retention time (RT) =35.706 (**b**), Limonen-6-ol, pivalate with RT = 36.06 (**c**), β- Himachaleoxide with RT = 36.39 (**d**), 4-tert-Butyltoluene with RT = 36.853 (**e**), α-Bisabolol with RT = 37.276 (**f**), Cholestan-3-ol, 2-methylene-, (3β, 5α) with RT = 36.325 (**g**), Benzenepropanol, 2, 4, 6-trimethyl- with RT =36.942 (**h**), and Z-(13,14-Epoxy) tetradec-11-en-1-ol acetate with RT = 36.613 (**i**).

**Figure 4:**
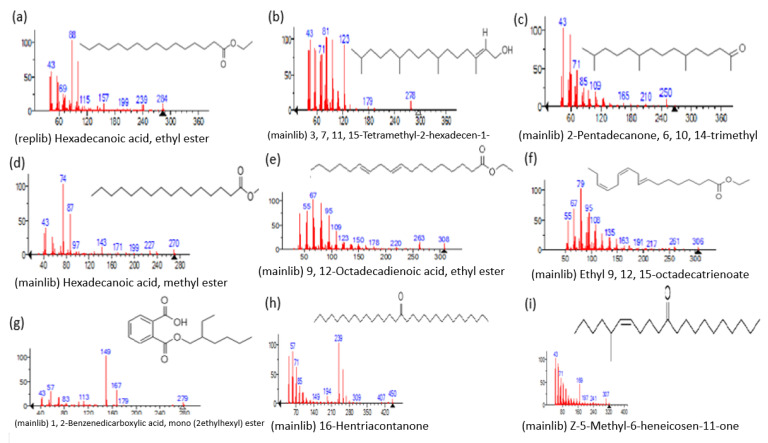
Representing the mass spectrum of Hexadecanoic acid, ethyl ester with RT = 41.613 (**a**), 3, 7, 11, 15 -Tetramethyl-2-hexadecen-1-ol with RT = 41.140 (**b**), 2-Pentadecanone, 6, 10, 14-trimethyl- with RT = 41.303 (**c**), Hexadecanoic acid, methyl ester with RT = 43.470 (**d**), 9,12-Octa decadienoic acid, ethyl ester with RT = 54.297 (**e**), Ethyl 9,12,15-octadecatrienoate with RT = 54.767 (**f**), 1,2-Benzenedicarboxylic acid, mono(2-ethylhexyl) ester with RT = 80.831 (**g**), 16-Hentriacontanone with RT = 91.164 (**h**), and Z-5-Methyl-6-heneicosen-11-one with RT = 92.799 (**i**).

**Figure 5:**
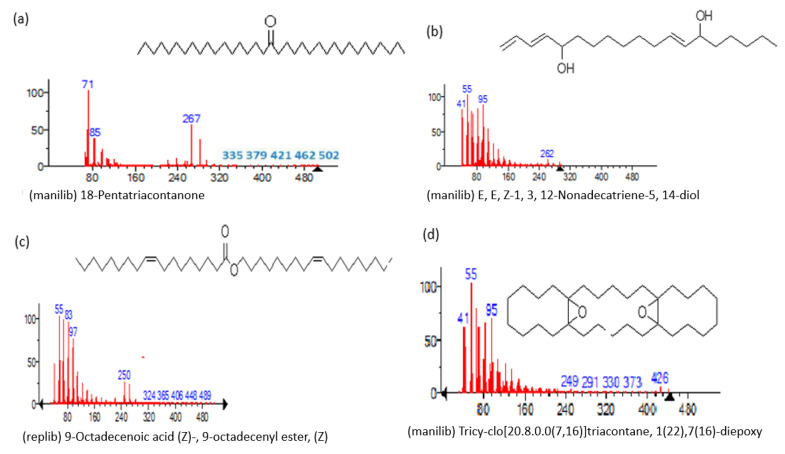
Representing the mass spectrum of 18-Pentatriacontanone with RT = 96.417 (**a**), E, E, Z-1, 3, 12-Nonadecatriene-5,14-diol with RT = 99.402 (**b**), 9-Octadecenoic acid (Z)-, 9-octadecenyl ester, (Z) - with RT = 103.82 (**c**), and Tricyclo [20.8.0.0(7, 16)] triacontane, 1(22), 7(16)-diepoxy- with RT = 104.247 (**d**).

The mass spectrum of the identified compounds is presented in Figure 3, Figure 4 and Figure 5.

**Figure 6:**
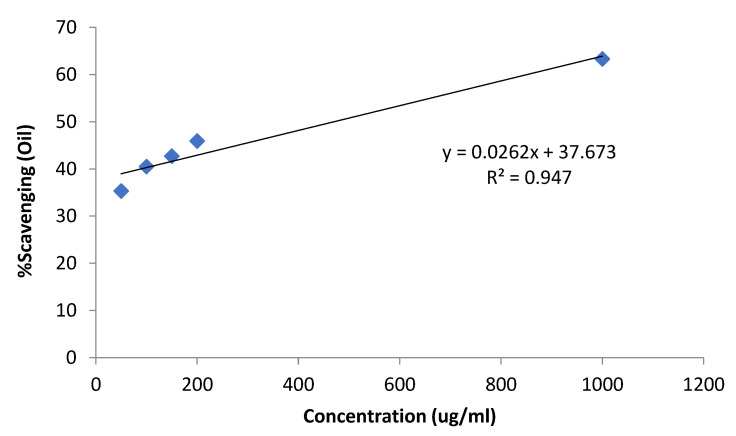
Equation to determine the ${IC}_{50}$ values of essential oil.

In the antioxidant assay (Table 2), concentrations were taken in (µg/mL), starting from 50, 100, 150, 200, and 1000 and shows the ${IC}_{50}$ values in Figure 6. The values of the essential oil that were obtained are 39.3, 40.5, 42.7, 45.9, and 63.3, while the values of the standard drug (ascorbic acid) are 70.5, 73.7, 77.8, 79.4, and 87.4, based on increasing concentrations. The *U. dioica* essential oil showed significant DPPH scavenging activity, as compared to the standard’s ascorbic acid. The essential oil of *U. dioica,* at low concentration, showed no growth inhibition, while at higher concentration, it showed inhibition power. Similar activity was reported by Kukri et al., using crude extract [39]. In a recent study conducted by Chaqroune and Taleb (2022), the findings showed significant antioxidant effects of the essential oil. The methanol and ethanol were used as solvents for extraction [40,41]. These findings may correlate to this study, as essential oils have the potential to be used as antioxidant agents.

In the phytotoxic assay (Table 3), the concentrations were taken in µg/mL, starting from 10, 100, 250, 500 and 1000 µg/mL. The concentration of the standard drug (0.015 µg/mL) was used. The percentage of the growth inhibition of the essential oil showed 26% and 62.5% at the concentrations of 500 and 1000 µg/mL, respectively, while at other concentration levels, showed a 0% result.

Table 4 contains the antibacterial results against five bacteria, *E. coli, B. subtilis, S. aureus, S. aeruginosa,* and *S. typhi*. Although the essential oil did not respond to the concentration of 250 μg/mL, it started to exhibit antibacterial activity against *E. coli* and *B. subtilis* at the concentration of 500 μg/mL, with an inhibition zone diameter of 10 ± 0.25 mm and 12 ± 0.32 mm. The antibacterial activity exhibited by the essential oil of *U. dioica* against the tested bacterial strains, *E. coli*, *B. subtilis*, *S. aureus*, *S. aeruginosa,* and *S. typhi,* showed good antibacterial activity (1000 μg/mL) as compared to the reference drug, ofloxacin (0.25 µg/mL). These findings concluded that the *U. dioica* essential oil could be used as a potential antibacterial agent for aromatherapy or topical applications against *E. coli* and *B. subtilis.* In addition, previous studies have also been reported on the antibacterial effects of *U. dioica* extract against selected pathogens [42,43]. The antibacterial activities of essential oils against the group of bacteria (*E. coli*, *B. subtilis*, and *S. aureus*) has been proven by Zeroual et al., 2021 [44]. Similarly, the current findings also showed a significant outcome against *E. coli*, *B. subtilis*, *S. aureus*, *S. aeruginosa,* and *S. typhi* bacteria.

### Computational Analysis

**Figure 7:**
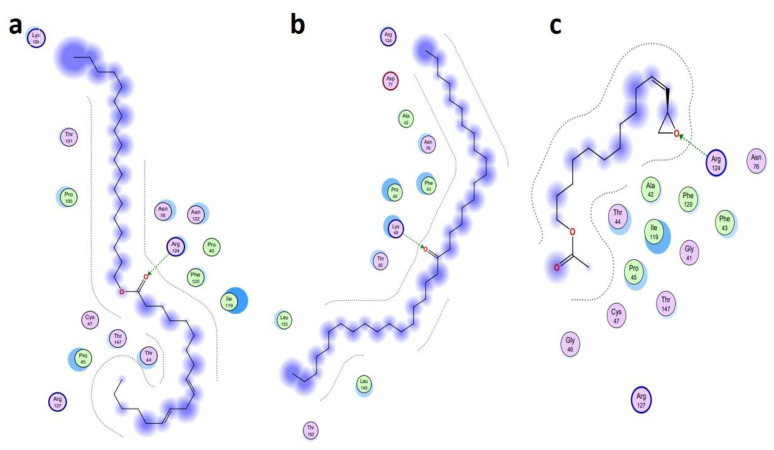
Interaction plot of top three scored docked ligands CID_ 22287839 (**a**), CID_10440 (**b**), and CID_ 5363633 (**c**), within active site of antioxidant protein [PDB ID: 1HD2], in the vicinity of 4Å.

**Figure 8:**
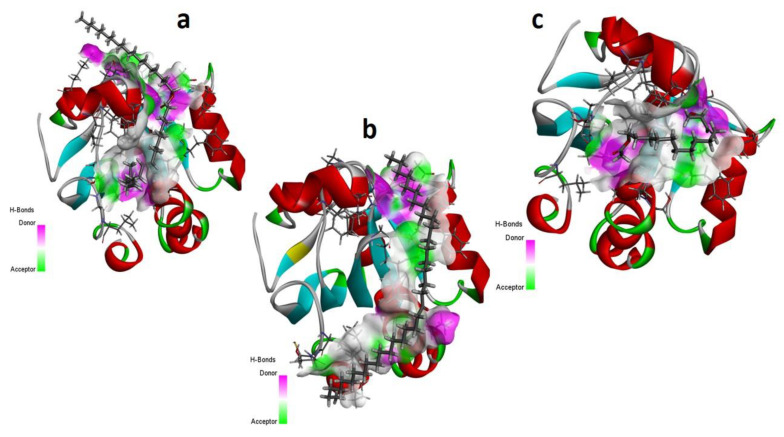
Hydrogen bonding capacity of top three scored docked ligands CID_ 22287839 (**a**), CID_10440 (**b**), and CID_5363633 (**c**), within the active site of antioxidant protein [PDB ID: 1HD2]. Purple colored area presents the hydrogen bond donor capacity and the green colored area presents the hydrogen bond acceptor capacity.

**Figure 9:**
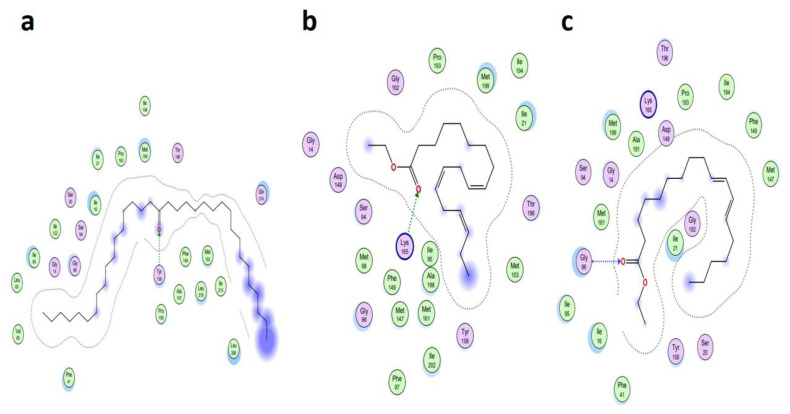
Interaction plot of top three scored docked ligands CID_10440 (**a**), CID_5367460 (**b**), and CID_22371644 (**c**), within active site of antibacterial protein [PDB ID: 4TZK], in the vicinity of 4Å.

**Figure 10:**
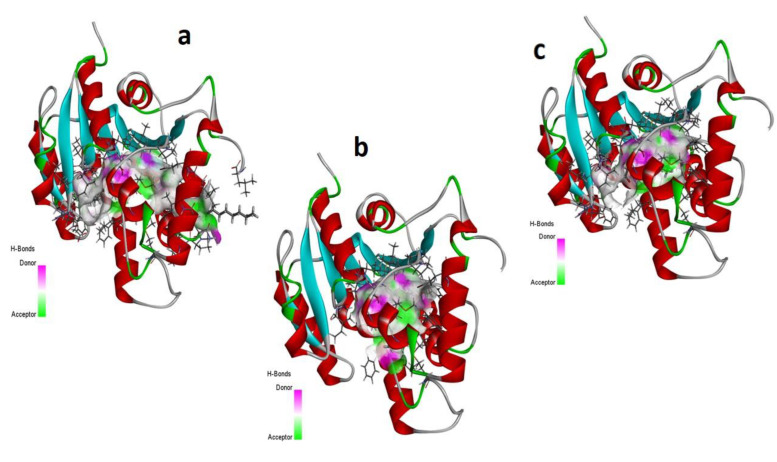
Hydrogen bonding capacity of top three scored docked ligands CID_10440 (**a**), CID_5367460 (**b**), and CID_22371644 (**c**), within the active site of antibacterial protein [PDB ID: 4TZK]. Purple colored area presents the hydrogen bond donor capacity and the green colored area presents the hydrogen bond acceptor capacity.

The MD investigations of the selected ligands with two target proteins were performed by MOE software. The binding energies of the selected ligands within the best binding pose were studied and it was observed that four ligands presented good results in terms of their binding interactions and binding energies in kcal/mol. A total of two best- bounded ligands in the vicinity of the active binding site of both the target proteins are shown in Figure 7, Figure 8, Figure 9 and Figure 10. The library of the 23 compounds was docked, and the three top virtual hits, with the antioxidant protein [PDB ID: 1HD2] [28] and antibacterial protein [PDB ID: 4TZK] [29] from the MD results, significantly presented a correlation with the experimental analysis of the whole *Urtica dioica* L. plant’s essential oil compounds. Hence, the two-dimensional interaction plots and three-dimensional presentation of the hydrogen binding pocket are shown in Figure 7, Figure 8, Figure 9 and Figure 10.

Table 5 presents the summary of the interaction analysis, the binding energies of the best-bounded conformation of the ligands with the target proteins in kcal/mol, and the interaction plot of top three best-docked ligands. 9-Octadecenoic acid (Z)-, 9-octadecenyl ester, (Z) [CID_22287839] presented the best-bounded conformation at −6.1991 kcal/mol, 18-Pentatriacontanone [CID_10440] presented the best-bounded conformation at −5.7512 kcal/mol, and Z-(13, 14-Epoxy) tetradec-11-en-1-ol acetate [CID_5363633] presented the best-bounded conformation at −5.2222 kcal/mol, within the active site of the antioxidant protein [PDB ID: 1HD2] and in the vicinity of 4Å (Figure 7 and Figure 8). Each color presents the type of interacting residues, and the hydrogen bonds are highlighted in each figure. In the interaction plot of the top three best-docked ligands, 18-Pentatriacontanone [CID_10440] presented the best-bounded conformation at −8.2366 kcal/mol, Ethyl 9, 12, 15-octadecatrienoate [CID_5367460] presented the best-bounded conformation at −7.8228 kcal/mol, and 9, 12-Octadecadienoic acid, ethyl ester [CID_22371644] presented the best-bounded conformation at −7.7674 kcal/mol, within the active site of the antibacterial protein [PDB ID: 4TZK] and in the vicinity of 4Å (Figure 9 and Figure 10).

The reported in silico applied protocols highlighted the standing of the ADMET profile for the short-listing of large chemical libraries, in order to justify the potential drug-like hits that can be tolerable in the design and development of novel drugs [34]. The ADMET justifications with the SwissADME and Datawarrior tools validated the properties of the selected hits, such as molecular weight (MW), partition coefficient/lipophilic parameters (logP values), hydrogen bond acceptor (HBA), hydrogen bond donor (HDB), total polar surface area (TPSA), molar refractivity (MR), and rotatable bond (RB), which are vital drug-like characteristics that are considered for the selected hits. As preliminary drug discovery protocols, they endorse estimations of drug-likeness, water solubility, pharmacokinetics, and toxicity estimations, along with medicinal chemistry perceptions, as highlighted for the selected potential hits in Table 6.

Not all the physicochemical properties of the virtual hits are in the acceptable range, and have some violations of Lipinski [42] and Veber’s [43] theory of drug-likeness because of a large MW. The lipophilicity and water solubility class also presented very good outcomes for Z-(13, 14-Epoxy) tetradec-11-en-1-ol acetate, Ethyl 9, 12, 15-octadecatrienoate, and 9, 12-Octadecadienoic acid, ethyl ester. The gastrointestinal drug absorption (GI-DA) [44] and blood–brain barrier (BBB) permeability [44] were also calculated for the selected five hits. The CYP1A2, CYP2C19, CYP2C9, CYP2D6, and CYP3A4 inhibitory potential was estimated, and it was observed that Z-(13, 14-Epoxy) tetradec-11-en-1-ol acetate, Ethyl 9, 12, 15-octadecatrienoate, and 9, 12-Octadecadienoic acid, ethyl ester is CYP1A2 and CYP2C9 inhibitors, although both compounds, 9-Octadecenoic acid (Z)-, 9-octadecenyl ester, (Z) and 18-Pentatriacontanone are P-glycoprotein (P-gp) substrates. The Log Kp (skin permeation) values were high for all the hits, and the PAINS alert and Brenk alert that were supported by the medicinal chemistry parameter evaluation [30] showed minor violations, which provides suggestions to improve the structures’ functionality and activity before moving a drug to the next phase of development. Synthetically, all the hits were highly accessible, with high scores, although minor toxicity was presented for one hit, Z-(13, 14-Epoxy) tetradec-11-en-1-ol acetate. Hence, the selected hits presented moderate drug-like activities.

## 4. Conclusions

The present study was conducted on *U. dioica* essential oil, which resulted in the identification of 22 bioactive compounds. For this identification, a GC–MS analysis was carried out. However, this study was not conducted with an individual compound. The whole *U. dioica* plant’s essential oil revealed potential antioxidant and antimicrobial activities in this study, potentially for the first time using the GC–MS-derived bioactive compounds of the *U. dioica* plant’s essential oil. The most significant outcome of this study was to reveal the phytotoxic effect of these essential oils for the first time. Moreover, future research could surely contribute to the selection of an individual compound obtained from the essential oil of *U. dioica*. The MD investigations assisted the experimental studies with respect to the protein–ligand binding conformation representations, in terms of the binding affinity and docked score, and helps in understanding the mechanism, while the ADMET estimation helps to justify the drug-like characteristics. Hence, these hits are recommended for clinical investigations, and it is expected that, in drug development, our results on antioxidant and antibacterial hits could surely contribute to the selection of significant drug candidates obtained from the essential oil of *U. dioica*.

## Figures and Tables

**Table 1:** Phytochemical compounds identified from *U. dioica* essential oil of DCM fraction.

Name of Compound	Mass/RT (min)	% Area	Molecular Formula	Fragments Ions	Structure
Nonanoic acid, 9-oxo-, ethyl ester	200/33.69	2.32	C11H20O3	41,55,88,29,43,101,83,60,157,155	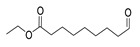
Carophyllen oxide	220/35.70	0.28	C15H24O	43,41,79,93,91,95,69,55,67,81	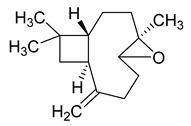
Limonen-6-ol, pivalate	236/36.06	0.23	C15H24O2	57,41,43,93,55,107,109,91,85,119	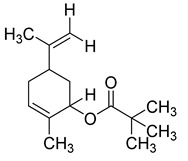
β-Himachalenoxide	220/36.39	0.45	C15H24O	110,220,95,192,43,41,69,109,151,93	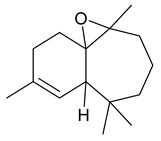
4-tert-Butyltoluene	148/36.85	0.4	C11H16	133,105,148,93,41,91,134,39,115,77	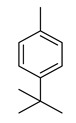
α-Bisabolol	222/37.27	1.25	C15H26O	109,119,69,43,93,41,95,121,67,71	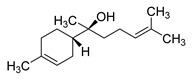
Cholestan-3-ol, 2-methylene-, (3β, 5α)-	400/36.32	0.18	C28H48O	69,81,71,95,67,83,79,93,97,105	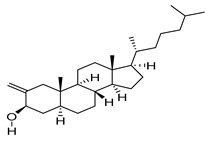
Benzenepropanol, 2, 4, 6-trimethyl-	178/39.84	0.17	C12H18O	133,178,134,120,119,91,145,105,117,115	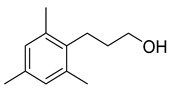
Z-(13, 14-Epoxy) tetradec-11-en-1-ol acetate	268/36.61	1.01	C16H28O3	43,97,69,55,41,82,67,81,84,83	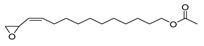
Hexadecanoic acid, ethyl ester,	284/40.11	0.47	C18H36O2	88,101,43,55,41,57,69,73,71,70	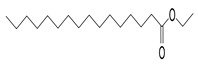
3, 7, 11, 15-Tetramethyl-2-hexadecen-1-ol	296/40.14	1.01	C20H40O	81,82,43,95,123,55,41,57,71,68	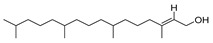
2-Pentadecanone, 6, 10, 14-trimethyl	268/41.30	1.76	C18H36O	43,58,71,57,59,41,55,69,85,95	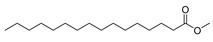
Hexadecanoic acid, methyl ester	270/43.47	0.66	C17H34O2	74,87,43,41,55,75,29,57, 143	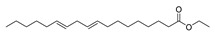
9, 12-Octadecadienoic acid, ethyl ester	308/54.29	1.92	C20H36O2	67,81,55,95,68,54,96,69	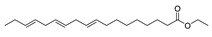
Ethyl 9, 12, 15-octadecatrienoate	306/54.76	3.33	C20H34O2	79,67,95,93,81,55,8o,107,91	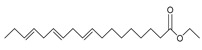
1, 2-Benzenedicarboxylic acid, mono (2-ethylhexyl) ester	278/80.83	5.12	C16H22O4	149,167,57,71,43,70	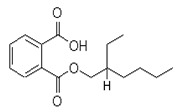
16-Hentriacontanone	450/91.16	1.52	C31H62O	239,57,43,71,255,55,58,245,69,41	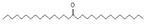
Z-5-Methyl-6-heneicosen-11-one	322/92.79	0.94	C22H42O	43,55,57,41,83,169,85,81,69	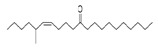
18-Pentatriacontanone	506/96.41	1.161	C35H70O	71,267,69,85,83,283,97,82	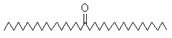
E, E, Z-1, 3, 12-Nonadecatriene-5, 14-diol	294/99.40	8.39	C15H26O2	55,95,81,41,67,69,96,83,43,57	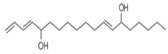
9-Octadecenoic acid (Z)-, 9-octadecenyl ester, (Z)	532/103.82	1.00	C36H68O2	55,69,83,97,82,96,57,95,67	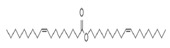
Tricyclo[20.8.0.0(7,16)]triacontane, 1(22),7(16)-diepoxy	444/104.24	0.42	C30H52O2	55,67,95,81,69,41,43,83,79,109	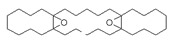

**Table 2:** Antioxidant activity of *U. dioica* essential oil.

Conc. (µg/mL)	%Scavenging (Essential Oil)	${IC}_{50}$ (Essential Oil)	%Scavenging (Ascorbic Acid)
50	35.3 ± 1.8		70.5 ± 2.3
100	40.5 ± 1.9		71.7 ± 3.1
150	42.7 ± 2.1	470.4	72.9 ± 2.0
200	45.9 ± 1.7		73.4 ± 2.4
1000	63.3 ± 1.8		87.4 ± 3.0

**Table 3:** Phytotoxic activity of *U. dioica* essential oil.

Conc. (µg/mL)	Essential Oil	Control	%Growth Inhibition	Std. Drug Conc. (µg/mL)
10	24	24	0	0.015
100	24	24	0	
250	24	24	0	
500	17	24	26	
1000	09	24	62.5	

**Table 4:** Antibacterial activity of *Urtica dioica* essential oil against selected bacteria strains.

Bacteria	*U. dioica* Essential Oil			Control (Ofloxacin) 0.25 µg/mL
	250 μg/mL	500 μg/mL	1000 μg/mL	
	Inhibition Zone Diameter (mm)			
*E. coli*	0	10 ± 0.25	32 ± 1.4	92.47 ± 2.3
*B. subtilis*	0	12 ± 0.32	35 ± 1.3	91 ± 3.1
*S. aureus*	0	0	25 ± 1.2	94 ± 2.4
*P. aeruginosa*	0	0	26 ± 1.1	94 ± 3.1
*S. typhi*	0	0	20 ± 1.5	95 ± 2.3

**Table 5:** Summary of interaction analysis of the two best virtual hits.

Ligand Name	Binding Energy (Kcal/mol)	Binding Interaction			
		Interacting Residues	Interaction Type	Bond Distance	Bond Energy (Kcal/mol)
Antioxidant protein [PDB ID: 1HD2]					
9-Octadecenoic acid (Z)-, 9-octadecenyl ester, (Z) [CID_22287839]	−6.1991	O2–NH2 ARG 124 (A)	H-acceptor	3.15	−2.0
18-Pentatriacontanone [CID_10440]	−5.7512	O1–NZ LYS 49 (A)	H-acceptor	2.92	−6.4
Z-(13, 14-Epoxy) tetradec-11-en-1-ol acetate [CID_5363633]	−5.2222	O1–NH2 ARG 124 (A)	H-acceptor	3.05	−0.5
Antibacterial protein [PDB ID: 4TZK]					
18-Pentatriacontanone [CID_10440]	−8.2366	O1–OH TYR 158 (A)	H-acceptor	2.93	−2.3
Ethyl 9, 12, 15-octadecatrienoate [CID_5367460]	−7.8228	O2–NZ LYS 165 (A)	H-acceptor	3.16	−1.1
9, 12-Octadecadienoic acid, ethyl ester [CID_22371644]	−7.7674	O2–N GLY 96 (A)	H-acceptor	3.33	−1.8

**Table 6:** Computational protocols applied for ADMET profile.

Chemical Parameters	9-Octadecenoic Acid (Z)-, 9-Octadecenyl Ester, (Z)	18-Pentatriacontanone	Z-(13, 14-Epoxy) tetradec-11-en-1-ol Acetate	Ethyl 9, 12, 15-Octadecatrienoate	9, 12-Octadecadienoic Acid, Ethyl Ester
Physicochemical Properties					
Molecular weight (MW) (g/mol)	532.92	506	268.39	306.48	308.50
Rotatable bonds	32	32	13	15	16
Hydrogen bond acceptors (HBA)	2	1	3	2	2
Hydrogen bond donors (HBD)	0	0	0	0	0
Molar Refractivity (MR)	175.50	170.56	78.81	98.12	98.59
Total polar surface area (TPSA) (Å)	26.30	17.07	38.83	26.30	26.30
Bioavailability Score	0.17	0.17	0.55	0.55	0.55
Lipophilicity					
Log Po/w (iLOGP)	8.73	8.68	3.44	4.82	5.01
Water Solubility					
Class	Insoluble	Insoluble	Soluble	Moderatley soluble	Moderatley soluble
Pharmacokinetics					
GI absorption	Low	Low	High	High	High
BBB permeant	No	No	Yes	No	No
P-gp substrate	Yes	Yes	No	No	No
CYP1A2 inhibitor	No	No	Yes	Yes	Yes
CYP2C19 Inhibitor	No	No	No	No	No
CYP2C9 inhibitor	No	No	Yes	Yes	Yes
CYP2D6 inhibitor	No	No	No	No	No
CYP3A4 inhibitor	No	No	No	No	No
Log Kp (skin permeation) (cm/s)	1.61	2.51	−4.74	−3.44	−2.79
Toxicity estimation					
Mutagenic	No	No	Yes	No	No
Tumorigenic	No	No	Yes	No	No
Reproductive effects	No	No	No	No	No
Irritant effects	No	No	Yes	No	No
Medicinal chemistry-related properties					
PAINS	No	No	No	No	No
Brenk	1 t: isolated_alkene	No	3: Three-membered_heterocycle, isolated_alkene	1: isolated_alkene	1: polyene
Synthetic accessibility	5.29	4.57	3.60	3.26	3.53

## Data Availability

Data will be available upon request. The data are not publicly available due to university research and management policy.
